# Assessing health-related quality of life in Japanese children with a chronic condition: validation of the DISABKIDS chronic generic module

**DOI:** 10.1186/s12955-018-0911-1

**Published:** 2018-05-02

**Authors:** Hatoko Sasaki, Naoko Kakee, Naho Morisaki, Rintaro Mori, Ulrike Ravens-Sieberer, Monika Bullinger

**Affiliations:** 1 Medical Support Center for Japan Environment and Children’s Study (JECS), National Center for Child Health and Development, 2-10-1 Okura, Setagaya, Tokyo, 157-8535 Japan; 2 Division of Bioethics, National Center for Child Health and Development, Tokyo, Japan; 3 Department of Social Medicine, National Center for Child Health and Development, Tokyo, Japan; 4 Department of Health Policy, National Center for Child Health and Development, Tokyo, Japan; 5 Department of Child and Adolescent Psychiatry, Psychotherapy, and Psychosomatics, University Medical Center Hamburg-Eppendorf, Hamburg, Germany; 6 Department of Medical Psychology, University Medical Center Hamburg-Eppendorf, Hamburg, Germany

**Keywords:** Children, Adolescents, Chronic conditions, Health-related quality of life, Assessment, Validation

## Abstract

**Background:**

This study examined the reliability and validity of the Japanese versions of the DISABKIDS-37 generic modules, a tool for assessing the health–related quality of life (HRQOL) of children and adolescents with a chronic condition.

**Methods:**

The study was conducted using a sample of 123 children/adolescents with a chronic medical condition, aged 8–18 years, and their parents. Focus interviews were performed to ensure content validity after translation. The classical psychometric tests were used to assess reliability and scale intercorrelations. The factor structure was examined with confirmatory factor analysis (CFA). Convergent validity was assessed by the correlation between the total score and the sub-scales of DISABKIDS-37 as well as the total score of KIDSCREEN-10.

**Results:**

Both the children/adolescent and parent versions of the score showed good to high internal consistency, and the test-retest reliability correlations were *r* = 0.91 or above. The CFA revealed that the modified models for all domains were better fit than the original 37 item scale model for both self-report and proxy-report. Moderate to high positive correlations were found for the associations within DISABKIDS-37 sub-scales and between the subscales and total score, except for the treatment sub-scale, which correlated weakly with the remaining sub-scales. The total score of the child-reported version of KIDSCREEN-10 correlated significantly and positively with the total score and all the sub-scales of the child-reported version of DISABKIDS-37 except the Treatment sub-scale in adolescents.

**Conclusions:**

The modified models of Japanese version of DISABKIDS generic module were psychometrically robust enough to assess the HRQOL of children with a chronic condition.

**Electronic supplementary material:**

The online version of this article (10.1186/s12955-018-0911-1) contains supplementary material, which is available to authorized users.

## Background

In 2013, there were 106,937 children (123,435 including registered patients receiving growth hormone therapy) with a chronic condition who received “Medical Aid for Specific Chronic Diseases of Children” in Japan [1]. The total number of registrations remained above 100,000 for the last five years [2]. Health–related quality of life (HRQOL) tends to be low in children with chronic conditions in Japan [3–5]. Factors include strains related to the management of the illness, increased frequency of hospital visits, and problems in life at school and at home [6, 7]. HRQOL is widely recognized as an important patient-reported outcome (PRO) in the clinical setting as it is critical for understanding the trajectories of health and illness, impacts of treatment, and issues of non-adherence in children with special health care needs [8]. Using such information, health professionals and teachers may be able to identify the types of intervention or educational support required to respond to children’s specific needs and to improve their HRQOL.

Although there is an increasing need for understanding the HRQOL in children and adolescents with a chronic condition in Japan, there are very few self-reported instruments designed to assess HRQOL specifically in this population. The Japanese version of PedsQL [9], KIDSCREEN [10, 11], and Kiddy-KINDLR [12] are available for pediatric HRQOL. The European DISABKIDS project [13] developed a pediatric instrument for measuring the HRQOL of children and adolescents aged 8–18 years with a chronic condition containing chronic generic- and disease-specific modules as well as a ‘smiley’ version for younger children aged 4–7 years. The development process across seven European countries resulted in cross-culturally valid, chronic, generic- as well as condition-specific DISABKIDS modules for both the self-reported and parent-reported versions. The DISABKIDS smiley measure for assessing HRQOL in younger children aged 4–7 years has already been validated for Japanese children with a chronic condition [14].

In the present study, the authors aimed to provide a Japanese version of the DISABKIDS chronic generic module and examine its reliability and validity for assessing the HRQOL of Japanese children and adolescents.

## Methods

### Ethical considerations

Participating in the pre-test (focus interviews) was voluntary, and written consent was obtained from all participants: verbal consent was obtained from children aged 8–18 years and the written consent form was completed by their parents. Participation in the field test was voluntary and written consent was forfeited since the survey was fully anonymized. All participants were assured of the anonymity and confidentiality of the data. The pre-test and field-test were approved by the Ethical Review Board of the National Center for Child Health and Development (NCCHD) in Tokyo, Japan on November 1, 2013 and December 27, 2013, respectively.

### Participants

We recruited chronically ill children and adolescents aged 8–18 years (with asthma, dermatitis, respiratory disease, endocrine disease, or collagen disease) and their families as outpatients of the NCCHD in Japan. NCCHD is the only national hospital to encompass the four areas of paediatric, perinatal, obstetric and maternal medicine in Japan. The hospital has 9 medical departments with 470 beds covering main areas in paediatrics and obstetrics. Approximately 1000 outpatients visit the hospital per day. The inclusion criteria for the participants were treatment of the same chronic condition for at least six months, voluntary consent, and regular visits to a doctor. The exclusion criteria were difficulty reading and completing the questionnaire and inability to provide voluntary consent. We aimed at a balanced distribution of gender and age.

### Procedure

#### Translation and pre-test (focus interviews)

Developing the instrument and testing the guidelines of the DISABKIDS group was followed by translating the questionnaire and conducting the pre-test [15]. After revising the forward-back translations, an international exchange with members of the DISABKIDS group was done to evaluate the final forward translation. Focus interviews were conducted with six male and female children (8–12 years old), and six male and female adolescents (13–18 years old), as well as with their parents using the Japanese translation. To test content validity, a researcher (interviewer) asked participants whether 1) each item was easy to understand, 2) any relevant issues were missing, and 3) the response scale agreed with each item, and recorded their answers. This process was done from October to December 2013.

#### Field-test

In total, 123 patients and one parent of each child were invited to participate in the survey. Research assistants explained the purpose and procedure. After both child and parent gave their consent, they were asked to answer the questionnaire while they waited to be seen by a doctor. After completing the questionnaire, the participants received a retest questionnaire to be returned by mail two weeks after the first administration.

### Instrument

#### DISABKIDS chronic generic module 37

The DISABKIDS Chronic Generic Module is a self-reported and parent-reported 37-item instrument with response options ranging from ‘never’ to ‘always.’ The subscales are: Independence, Emotion, Social inclusion, Social exclusion, Physical Limitation, and Treatment. Independence assessed autonomy and the degree to which children were living without impairments caused by their condition. Physical Limitation assessed functional limitations and perceived health status. Emotion addressed worries, concerns, anger, and problems. Social Inclusion dealt with the acceptance of others and positive social relationships whereas Social Exclusion dealt with stigma and the feeling of being left out. Treatment described the emotional impact of taking medication, receiving injections, applying local treatment, etc. [16]. The DISABKIDS Group [16] reported that Cronbach’s alpha reliabilities with Independence (α = 0.78), Emotion (α = 0.87), Social inclusion (α = 0.70), Social exclusion (α = 0.75), Physical Limitation (α = 0.73) and Treatment (α = 0.80) for child version, and with Independence (α = 0.85), Emotion (α = 0.90), Social inclusion (α = 0.78), Social exclusion (α = 0.80), Physical Limitation (α = 0.77) and Treatment (α = 0.85) for proxy version. The test-retest reliability at one month interval with Item intraclass correlation (ICC) with Independence (ICC = 0.74), Emotion (ICC = 0.82), Social inclusion (ICC = 0.79), Social exclusion (ICC = 0.81), Physical Limitation (ICC = 0.83) and Treatment (ICC = 0.71) dimensions in child scores and Independence (ICC = 0.77), Emotion (ICC = 0.76), Social inclusion (ICC = 0.77), Social exclusion (ICC = 0.83), Physical Limitation (ICC = 0.80) and Treatment (ICC = 0.73) facets in proxy scores.

#### KIDSCREEN-10

The KIDSCREEN-10 [17] is a 10-item, abbreviated version of the original KIDSCREEN-52 [18] using a 7-point scale ranging from never to always, designed to measure the physical, psychological, and social aspects of quality of life in children and adolescents 8 to 18 years of age. Cronbach’s alpha reliabilities were 0.82 for the self-report (8–11 years old: 0.79, 12–18 years old: 0.81) and 0.78 for the proxy report (8–11 years old: 0.78, 12–18 years old: 0.78). Test-retest reliabilities were 0.70 for the self-report (8–11 years old: 0.64, 12–18 years old: 0.69) and 0.67 for the proxy report (8–11 years old: 0.64, 12–18 years old: 0.66). The Japanese version of KIDSCREEN-52 was developed and validated by Nezu et al. [10], who also reported on the reliability and validity of KIDSCREEN-10 [11]. In this study, the KIDSCREEN-10 was administered to test the convergent validity of the DISABKIDS Chronic Generic Module.

### Statistical analysis

All statistical analyses were performed using SPSS version 21.0 (IBM Corporation, USA). The distribution characteristics of each item were checked by the means and standard deviations (SDs). Skewness and the percentage frequency of the minimum and maximum values were calculated as the ceiling and floor effects, respectively. Internal consistency of the scales was tested using Cronbach’s alpha as well as Guttman split-half reliability. The item-subtotal correlation of Cronbach’s alpha and Cronbach’s alpha if item is deleted were calculated separately for the self-reported and parent-reported versions. Items with item-subtotal correlation < 0.3 [19], and within each subscale the items whose deletion would lead to an increase of alpha of at least 0.02 were candidate for deletion [13]. ICC were computed to assess the level of concordance between the self- and parent-reported scores. Test-retest reliability was assessed at a two-week interval using ICC to detect any systematic errors [20].

The factor structure of the Japanese DISABKIDS Chronic Generic Module was examined with confirmatory factor analysis (CFA) using AMOS version 21.0 (IBM Corporation, USA). As the original scale development and pilot study [21] performed the CFA separately for the Mental, Social and Physical in order to assess item loadings on each of these domains, we followed this approach. The values of goodness of fit (GFI) above 0.90 are common in adequate models, and values below 0.90 indicate poorly fit models. The comparative fit index (CFI) is considered good fit at values near one and poor fit at values below 0.90. The Root Mean Square Error of Approximation (RMSEA) is generally well fit models at values of 0.05 or less, and unusable models at values greater than 0.10 [22].

Pearson coefficients were computed to evaluate the intercorrelation between the sub-scales and total score. The convergent validity of the DISABKIDS scale was assessed based on its correlation with KIDSCREEN-10. We hypothesized that KIDSCREEN-10 would strongly correlate with the Japanese DISABKIDS Chronic Generic Module. We divided the age groups into the 8 to 12-year-old and the 13 to 18-year-old groups as in the original protocol for developing the DISABKIDS Chronic Generic Module. The discriminant validity was tested examining differences in HrQOL across gender, age, economic status and clinical characteristics.

## Results

### Translation and pre-test (focus interviews)

The translation process was completed in accordance with relevant guidelines, with two forward translations, reconciliation, and one backward translation as well as comparisons between the original and back translation [15]. With regard to difficulties in understanding the items, two children and four parents made comments and suggestions during the focus interviews. For example, a 10-year-old girl mentioned that “lonely” in one question should be written in hiragana (the Japanese cursive syllabary), which is easier for young children to read. A mother of a 10-year-old girl stated that she was not sure whether “frustrated” in one item referred to the frustration caused “by the disease itself” or “by symptoms of the disease”. Regarding the difficulties of using the response scale, two children and three parents made comments and suggestions. For example, a 15-year-old boy felt that there was a mismatch between some of the questions and response options. Hiragana was added to the term “lonely” as a help to read the latter for young children. We found that there was no need to omit any of the items from the scales or to add new concepts pertaining to HRQOL.

### Field test

#### Description of sample

Table 1 shows the descriptive statistics of the study sample including the number of socio-demographic and clinical variables. A total of 123 children with a chronic condition and 123 parents/caregivers completed the test and retest versions. The age of the children ranged from 8 to 18 years with 51.2% aged 8–12 years and 48.8% aged 13–18 years. The mean age of the parents was 45 years. More than half of the children had allergies, and a quarter had an endocrine disease.

**Table 1:** Socio-demographic and clinical characteristics of the sample

	Children/adolescents (*N* = 123)	Parents/caregivers (N = 123)
Age years, Mean (SD)	12.3 (3.1)	45.0 (5.5)
Age group, n (%)		
Children (8–12 years)	63 (51.2)	
Adolescents (13–18 years)	60 (48.8)	
Gender		
Male	66 (53.7)	16 (13.0)
Female	57 (46.3)	103 (83.7)
Unanswered		4 (3.3)
Marital status: married, n (%)		111 (90.2)
Economic status (living condition), n (%)		
Very comfortable		6(4.9)
Somewhat comfortable		43(35)
Somewhat difficult		60(48.8)
Very difficult		11(8.9)
Unanswered		3(2.4)
Clinical characteristics, n (%)		
Allergies	70 (56.9)	
Endocrine Disease	25 (20.3)	
Circulatory disease	13 (10.6)	
Kidney disease	11 (8.9)	
Collagen Disease	4 (3.3)	

#### Item analysis and reliability

The distribution characteristic of the scores for the child- and parent-reported versions are given in Table 2. Floor effects were rare while moderate ceiling effects were observed in three out of six dimensions (Social exclusion, Physical, and Treatment). The internal consistency reliabilities ranged from *r* = 0.92 (child-reported version) to *r* = 0.95 (parent-reported version). The Guttman split-half reliability reached a coefficient of *r* = 0.84 for the child-reported version and *r* = 0.85 for the parent-reported version. High levels of agreement between the child and parent reports were observed for both sub-scales (ICC = 0.64–0.74) and the total scores (ICC = 0.77). A high test–retest reliability was found across two time points for children (ICC for total score = 0.90, *p* < 0.001), adolescents (ICC for total score = 0.94, p < 0.001), and parents (ICC for total score = 0.96–0.97, p < 0.001) as shown in Table 3. The mean values, standard deviations, item-subtotal correlations and Cronbach’s alpha coefficients for all the 37 items are presented in Additional file 1. Item 6, 19 and 32 for child report had item-subtotal correlations lower than 0.3, and the deletion of these three items increased the Cronbach’s alpha of the subscales at least 0.02. Item 6, 19 and 32 for child data were dropped for the following analysis.

**Table 2:** Psychometric properties for the total score of the Japanese DISABKIDS Chronic generic module (self-report version / parent version)

	Domain	Facet	N	Mean	SD	%floor	%ceiling	Skewness	Cronbach α	Split-half	ICC
DISABKIDS	Mental	Independence	123/122	73.1/70.6	16.9/18.2	0.0/0.0	3.3/6.6	− 0.7/− 0.5	0.74/0.86	0.74/0.81	0.71/−
		Emotion	123/123	74.8/66.3	19.4/20.4	0.0/0.8	8.1/4.9	−0.7/−0.7	0.82/0.92	0.84/0.91	0.70/−
	Social	Social inclusion	123/122	73.3/72.2	20/18.5	0.0/0.0	9.8/8.2	−1.0/−0.6	0.78/0.80	0.81/0.86	0.70/−
		Social exclusion	122/123	81.2/74.9	18.1/19.4	0.0/0.0	20.3/11.4	−1.3/−0.8	0.75/0.87	0.71/0.83	0.74/−
	Physical	Physical	121/123	73.1/67.4	21.8/20.8	0.0/0.0	10.7/5.7	−0.8/− 0.5	0.79/0.86	0.75/0.85	0.73/−
		Treatment	110/113	74.9/62.3	20.7/21.7	0.0/0.0	18.2/4.4	−0.6/−0.4	0.77/0.88	0.68/0.87	0.64/−
Total score			123/123	75.1/70.2	16/16.2	–	–	−1.1/−0.6	0.92/0.95	0.84/0.85	0.77/−

ICC: Infraclass correlation coefficients (ICC) between self-report version and proxy-report version of the Japanese DISABKIDS measure

**Table 3:** Test-retest reliability correlation (self-report version / parent version)

	Domain	Facet	Children (*n* = 63)		Adolescents (*n* = 60)	
			Pearson r	ICC	Pearson r	ICC
DISABKIDS	Mental	Independence	0.69/0.78	0.81/0.87	0.83/0.81	0.90/0.89
		Emotion	0.65/0.83	0.78/0.90	0.82/0.86	0.90/0.92
	Social	Social inclusion	0.79/0.88	0.88/0.93	0.83/0.92	0.91/0.96
		Social exclusion	0.75/0.89	0.85/0.94	0.74/0.82	0.85/0.90
	Physical	Physical limitation	0.47/0.84	0.64/0.90	0.84/0.81	0.91/0.89
		Treatment	0.68/0.87	0.81/0.93	0.74/0.82	0.85/0.93
Total score			0.83/0.94	0.90/0.97	0.89/0.93	0.94/0.96

All correlations are significant, *P* < 0.01

#### Confirmatory factor analysis

The summary of CFA was shown in Additional file 2. The original 12-item Mental domain for the child reported data (Model 0) had low goodness of fit values: GFI = 0.874, CFI = 0.924, and RMSEA = 0.074. After deletion of Item 6 and 19, the 10-item Mental domain (Model 1) had also low goodness of fit values: GFI = 0.894, CFI = 0.942, and RMSEA = 0.078. We therefore added the error covariance to the 10-item Mental domain model (Model 2) by using modification indices in Amos version 21.0, and the goodness of fit improved to GFI = 0.933, CFI = 0.992, and RMSEA = 0.030. The Social domain (Model 0) had low goodness of fit values: GFI = 0.871, CFI = 0.877, and RMSEA = 0.090. The error covariance correction model for Social domain (Model 1) had goodness of fit values with GFI = 0.902; CFI = 0.956; and RMSEA = 0.055. The original 12-item Physical domain (Model 0) had values with GFI = 0.892; CFI = 0.941; and RMSEA = 0.063. After deletion of Item 32, the 11-item Physical domain (Model 1) had values with GFI = 0.896; CFI = 0.936; and RMSEA = 0.073. The 11-item Physical domain added the error covariance (Model 2) improved values with GFI = 0.916; CFI = 0.977; and RMSEA = 0.044. The Mental domain for the proxy reported data (Model 0) had values with GFI = 0.856; CFI = 0.935; and RMSEA = 0.096. The Mental domain model with error covariance (Model 1) improved values with GFI = 0.909; CFI = 0.984; and RMSEA = 0.049. The Social domain (Model 0) had low goodness of fit values with GFI = 0.824; CFI = 0.875; and RMSEA = 0.124. The Social domain model with error covariance (Model 1) had acceptable fit of GFI = 0.884; CFI = 0.953; and RMSEA = 0.077. The Physical domain (Model 0) had low goodness of fit values with GFI = 0.824; CFI = 0.887; and RMSEA = 0.116. The Physical domain model with error covariance (Model 1) had an acceptable fit of GFI = 0.870; CFI = 0.931; and RMSEA = 0.093.

#### Intercorrelations between sub-scales and total score

Table 4 shows moderate to strong positive correlations among the sub-scales and between the subscales and total scores except for the Treatment sub-scale, which correlated weakly and/or negatively with the remaining sub-scales. No difference was observed in the correlations when the scores of 0 and 100 were excluded from the analysis (see Additional file 3).

**Table 4:** Intercorrelation (Pearson r) between total score and sub-scales of the self-report version and parent version

Self-report/ proxy-report						
	Independence	Emotion	Social inclusion	Social exclusion	Physical limitation	Treatment
Children (n = 63)						
Emotion	0.61**/0.61**					
Social inclusion	0.60**/0.57**	0.57**/0.28*				
Social exclusion	0.65**/0.59**	0.70**/0.69**	0.63**/0.42**			
Limitation	0.56**/0.61**	0.71**/0.71**	0.60**/0.39**	0.73**/0.76**		
Treatment	0.08/0.05	0.34*/0.53**	−0.06/−0.06	−0.01/0.45**	0.16/0.39**	
Total score	0.77**/0.75**	0.89**/0.87**	0.78**/0.55**	0.84**/0.86**	0.87**/0.86**	0.34*/0.59**
Adolescents (n = 60)						
Emotion	0.71**/0.74**					
Social inclusion	0.58**/0.54**	0.485**/0.31*				
Social exclusion	0.58**/0.61**	0.64**/0.62**	0.49**/0.63**			
Limitation	0.62**/0.71**	0.66**/0.80**	0.60**/0.54**	0.68**/0.77**		
Treatment	−0.04/0.47**	0.10/0.65**	0.09/0.16	0.19/0.33*	0.17/0.40**	
Total score	0.79**/0.85**	0.81**/0.88**	0.73**/0.66**	0.79**/0.83**	0.88**/0.90**	0.32*/0.65**
Cross-cultural sample^a^						
Emotion	0.52/0.55					
Social inclusion	0.57/0.60	0.40/0.49				
Social exclusion	0.49/0.55	0.57/0.68	0.46/0.55			
Limitation	0.41/0.42	0.62/0.64	0.42/0.34	0.36/0.42		
Treatment	0.31/0.33	0.60/0.55	0.24/0.30	0.44/0.49	0.37/0.36	
Total score	0.72/0.73	0.86/0.88	0.67/0.70	0.73/0.81	0.72/0.71	0.71/0.70

^a^The DISABKIDS Group Europe, *n* = 1152, age group 8–16. (Schmidt et al., [16])

*P < 0.05, **P < 0.01

#### Criterion and discriminant validity

A significant and positive correlation was found between the total score and sub-scale of the DISABKIDS-37 child-reported version, and the total score of the child-reported KIDSCREEN-10, except for a low association between the Treatment sub-scale and KIDSCREEN-10 in adolescents (*r* = 0.08, *p* = 0.544). Statistically significant associations were also observed between the total score and sub-scale of the parent-reported version of DISABKIDS-37 and the parent-reported version of KIDSCREEN-10, supporting convergent validity (see Table 5). No difference was observed in the correlation coefficients when 0 and 100 scores were excluded from the analysis (see Additional file 4).

**Table 5:** Correlation coefficients (Pearson r) for total score and sub-scales of the self-report and parent versions with KIDSCREEN (self-report and parent versions)

		Independence	Emotion	Social inclusion	Social exclusion	Physical limitation	Treatment	Total score
KIDSCREEN	Children							
	Self-report version	0.44**	0.54^#^	0.48^#^	0.38**	0.37**	0.41**	0.60^#^
	Parent version	0.55^#^	0.55^#^	0.59^#^	0.56^#^	0.52^#^	0.39**	0.68^#^
	Adolescents							
	Self-report version	0.65^#^	0.50^#^	0.65^#^	0.60^#^	0.61^#^	0.08	0.71^#^
	Parent version	0.67^#^	0.48^#^	0.58^#^	0.54^#^	0.61^#^	0.41**	0.68^#^
	Cross-cultural sample^a^ (self/proxy)	0.70/0.79	0.84/0.92	0.68/0.74	0.77/0.83	0.70/0.79	0.80/0.85	0.91/0.94

^a^The DISABKIDS Group Europe, *n* = 1152, age group 8–16 (Schmidt et al., [16])  *p < 0.05, **p < 0.01, ^#^*p* < 0.001

Comparisons of the dimension scores for self-report version according to gender, age group, economic status and clinical characteristics were shown in Table 6. There was no difference in HRQOL in gender and clinical characteristics. Older adolescents reported lower emotional and physical well-being than younger children. Children belonging to families with lower level of economic status were found to have worse HRQOL in independent, emotion and social inclusion dimensions than children belonging to families with higher level of economic status.

**Table 6:** Comparisons of the dimension scores for self-report version according to gender, age group, economic status and clinical characteristics

	Independence	Emotion	Social inclusion	Social exclusion	Physical limitation	Treatment	Total score
Gender							
Boys	74.2 ± 18.9	75.1 ± 21.3	73.5 ± 20.0	80.4 ± 18.7	74.1 ± 20.7	74.3 ± 24.9	74.5 ± 15.2
Girls	75.5 ± 17.9	78.1 ± 19.7	74.3 ± 17.9	82.1 ± 17.5	71.8 ± 23.1	71.9 ± 20.9	74.8 ± 15.6
P values	0.68	0.42	0.81	0.62	0.55	0.59	0.92
Age group							
Children	77.8 ± 17.5	81.2 ± 19.3	74.8 ± 18.2	82.3 ± 19.8	78.2 ± 20.7	76.4 ± 22.3	77.8 ± 15.1
Adolescents	71.6 ± 19.0	71.5 ± 20.9	72.9 ± 19.8	79.9 ± 16.3	67.8 ± 21.8	70.0 ± 23.3	71.5 ± 15.0
P values	0.06	0.01	0.57	0.46	0.01	0.14	0.03
Economic status^a^							
Comfortable	79.6 ± 15.8	81.8 ± 16.3	77.5 ± 14.7	84.3 ± 16.5	75.0 ± 18.3	74.6 ± 21.8	78.4 ± 12.4
Difficult	71.3 ± 19.4	72.5 ± 22.5	71.1 ± 21.2	78.8 ± 18.9	71.6 ± 23.9	72.2 ± 23.7	72.3 ± 16.5
*P* values	0.01	0.01	0.05	0.09	0.38	0.60	0.04
Clinical characteristics^b^							
Allergies	77.0 ± 18.4	79.3 ± 20.0	75.6 ± 19.0	80.8 ± 19.1	76.2 ± 21.2	74.8 ± 21.3	77.4 ± 15.2
Endocrine Disease	68.2 ± 20.6	69.0 ± 21.9	70.3 ± 20.2	76.0 ± 18.8	65.0 ± 23.8	66.3 ± 27.0	68.8 ± 15.8
Others	75.2 ± 15.4	75.9 ± 19.9	72.7 ± 17.8	87.0 ± 13.0	72.6 ± 20.1	75.6 ± 23.4	71.7 ± 13.5
P values	0.12	0.10	0.46	0.09	0.09	0.28	0.06

Dimension scores are reported as mean standard deviation

^a^ Economic status is recorded into two categories: Comfortable includes “Very comfortable” and “Somewhat comfortable”. Difficult includes “Somewhat difficult” and “Very difficult”

^b^ *P* values: significance level of analysis of variance test. “Others” include Circulatory disease, Kidney disease, and Collagen Disease

## Discussion

The present study examined the psychometric properties of the DISABKIDS Chronic Generic Module for assessing HRQOL in Japanese children and adolescents with a chronic condition using both child- and parent-reported versions. Following the DISABKIDS group guidelines, we translated items and responses and examined their content through focus interviews. Our results revealed good internal consistency, split-half reliability, and test-retest reliability for the DISABKIDS Chronic Generic Module. Three items for child report data had item-subtotal correlations lower than 0.3, and the deletion of these three items increased the Cronbach’s alpha of the subscales at least 0.02. The CFA revealed that the modified models for all domains were better fit than the original 37 item scale model for both self-report and proxy-report. Good convergent validity of this scale with both the child-reported and parent-reported versions of KIDSCREEN-10 was observed. The results indicated that the modified models of Japanese version of DISABKIDS Chronic Generic Module are psychometrically acceptable for assessing the HRQOL of Japanese children and adolescents with a chronic condition.

Moderate ceiling effects in the Social exclusion, Physical, and Treatment dimensions were observed mainly in the self-report. This finding was not unique to our sample, as similar ceiling effects have been reported in a European study [13] as well as for other HRQOL scales designed for children and adolescents with a chronic condition [23]. While there was agreement between child-reported and parent-reported scores, the child’s rating of quality of life was higher than that of their parent for all facets as observed in the previous studies [16, 24].

Item 6, 19 and 32 for child report data had item-subtotal correlations lower than 0.3, and the deletion of these three items increased the Cronbach’s alpha of the subscales at least 0.02. This indicated that these three items may need to be rephrased. We suspected that the term “things” in item 6 “Are you able to do things without your parents?” was not specific and therefore it might be difficult to answer the question. Item 19 “Does it bother you that your life has to be planned?” and item 32 “Does having to get help with medication from others bother you?” contained the common term “bother”, which might have made it difficult to interpret.

The Japanese version of the DISABKIDS 37 showed weakness in terms of structural models. Translation or cultural aspects cannot be eliminated as a factor of error variation. However, the good to high level of internal consistency reliabilities indicated that reasons may lie elsewhere. Although a suitable structure for this measure verified in the cross-cultural validation study, there were some restrictions in several countries [16]. Therefore, some aspects of model unfitness in this study could be due to instabilities or inadequacies in the conceptual foundation of the construct itself.

We observed a moderate to strong, positive association among the DISABKIDS sub-scales and between the subscales and total scores [16] except for the Treatment sub-scale, which correlated weakly with the remaining sub-scales. The validation studies for the Portuguese version of DISABKIDS also found a weak association between the Treatment sub-scale and the other sub-scales [25]. On the other hand, the correlation coefficients between the Treatment sub-scale and the other sub-scales in children and adolescents were lower than those in their parent, which was not previously observed in other populations using the same scales. Cultural factors may have caused the divergence, i.e., in Japan children may tend to accept their condition and treatment so that the burden imposed by these may not impair other aspects of their life. Convergent validity was inferred from a statistically significant correlation between child-reported DISABKIDS-37 and KIDSCREEN-10. On a descriptive level, low emotional well-being was found in adolescents and in children belonging families with relatively low economic status. These findings have been also observed in previous studies [13].

The limitations of our study include the selectivity of the sample in terms of clinical conditions (mainly allergies and endocrine diseases) and its monocentric design (only one facility in Japan). A larger sample of different clinical conditions from a variety of facilities may have yielded different psychometric properties. Also, because of the cross-sectional study design, it was not possible to examine the sensitivity of the instrument for detecting changes in the children’s health condition. These challenges will need to be addressed in future studies.

This study validates the utility of the modified models of DISABKIDS Chronic Generic Module for assessing the HRQOL of Japanese children and adolescents with a chronic condition. It is particularly important not only for the children themselves but also for health professionals, teachers, parents, and other caregivers to understand the importance of HRQOL over the course of treatment. Moreover, with the many language versions available, DISABKIDS may be used in future, cross-cultural research comparing HRQOL.

## Conclusion

The findings of this study demonstrated acceptable psychometric performance of the modified models of DISABKIDS-37 when used with Japanese children and adolescents with a chronic condition. Further research in larger samples is needed to determine whether the same factors will be extracted and their validity can be verified. The HRQOL assessment used for this particular population may not only be of value in clinical trials of new therapies, but will facilitate communication between patients and health professionals, identify healthcare needs, and support life at home and school.

## Additional files

Additional file 1: Mean, SD, Correlations, and Cronbach α of Items. (PDF 121 kb)

Additional file 2: Summary of Confirmatory Factor Analysis. (PDF 10 kb)

Additional file 3: Intercorrelation (Pearson r) between total score and sub-scales for excluding 0 and 100 scores. (PDF 78 kb)

Additional file 4: Correlation coefficients (Pearson r) for total score and sub-scales with the KIDSCREEN for excluding 0 and 100 scores. (PDF 97 kb)

## Abbreviations

HRQOL: Health-related quality of life
ICC: Intraclass correlation coefficient
PRO: Patient-reported outcome
SD: Standard deviation
SPSS: Statistical package for social sciences
