# Supplementary material for: Assessing health-related quality of life in Japanese children with a chronic condition: validation of the DISABKIDS chronic generic module
Source: Health Qual Life Outcomes. 2018 May 2;16:85. doi: 10.1186/s12955-018-0911-1 (PMC5932858; doi:10.1186/s12955-018-0911-1)
Supplement: Supplementary file 1 — Mean, SD, Correlations, and Cronbach α of Items. (PDF 121 kb) [file 12955_2018_911_MOESM1_ESM.pdf]

Appendix 1: Mean, SD, Correlations, and Cronbach α of Items

	Subscale	Item	N	Mean	SD	Item-total correlation of Cronbach α	Cronbach α if item is deleted	Cronbach α of the subscale
Self-report	Independence	1. Are you confident about your future?	122	3.64	1.00	.39	.72	0.74
		2. Do you enjoy your life?	123	4.50	0.74	.44	.72	
		3. Are you able to do everything you want to do even though you have your condition?	123	4.02	1.06	.58	.67	
		4. Do you feel like everyone else even though you have your condition?	123	3.81	1.21	.55	.68	
		5. Are you free to lead the life you want even though you have your condition?	123	3.98	1.06	.70	.63	
		6. Are you able to do things without your parents?	123	3.59	1.07	**.24**	**.77**	
	Emotion	13. Does your condition make you feel bad about yourself?	123	4.26	0.97	.62	.79	0.82
		14. Are you unhappy because of your condition?	123	4.43	0.82	.64	.79	
		15. Do you worry about your condition?	123	3.63	1.12	.49	.81	
		16. Does your condition make you angry?	123	4.03	1.18	.69	.77	
		17. Do you have fears about the future because of your condition?	123	4.03	1.14	.66	.78	
		18. Does your condition get you down?	123	3.96	1.22	.70	.77	
		19. Does it bother you that your life has to be planned?	123	3.59	1.31	**.25**	**.85**	
	Social inclusion	26. Do other children/adolescents understand your condition?	122	3.72	1.30	.37	.79	0.78
		27. Do you go out with your friends?	123	3.95	1.09	.43	.76	
		28. Are you able to play or do things with other children/adolescents (like sports)?	123	4.28	1.12	.66	.71	
		29. Do you think that you can do most things as well as other children/adolescents?	123	4.02	1.19	.58	.73	
		30. Do your friends enjoy being with you?	123	4.39	0.89	.64	.73	
		31. Do you find it easy to talk about your condition to other people?	123	3.22	1.38	.54	.74	
	Social	20. Do you feel lonely because of your condition?	123	4.41	0.94	.41	.73	0.75
		21. Do your teachers behave differently towards you than towards others?	122	4.33	1.11	.43	.73	
		22. Do you have problems concentrating at school because of your condition?	122	4.36	0.99	.53	.70	
		23. Do you feel that others have something against you?	123	4.08	1.20	.58	.69	
		24. Do you think that others stare at you?	123	4.46	0.87	.37	.74	
		25. Do you feel different from other children/adolescents?	123	3.81	1.37	.62	.67	
	Physical	7. Are you able to run and move as you like?	122	4.44	1.00	.47	.78	0.79
		8. Do you feel tired because of your condition?	123	3.62	1.30	.63	.74	
		9. Is your life ruled by your condition?	122	4.04	1.20	.70	.72	
		10. Does it bother you that you have to explain to others what you can and can't do?	123	3.35	1.47	.40	.80	
		11. Is it difficult to sleep because of your condition?	120	4.23	1.13	.46	.78	
		12. Does your condition bother you when you play or do other things?	122	3.88	1.30	.66	.73	
	Treatment	32. Does having to get help with medication from others bother you?	113	4.36	1.10	**.23**	**.80**	0.77
		33. Is it annoying for you to have to remember your medication?	112	3.49	1.32	.65	.70	
		34. Are you worried about your medication?	112	4.31	0.98	.32	.78	
		35. Does taking medication bother you?	111	3.46	1.44	.69	.69	
		36. Do you hate taking your medicine?	110	4.11	1.27	.65	.70	
		37. Does taking medication disrupt everyday life?	111	4.28	1.07	.58	.73	
Proxy-report	Independence	1. Is your child confident about his/her future?	121	3.70	0.91	.46	.87	0.86
		2. Does your child enjoy his/her life?	122	4.28	0.71	.67	.84	
		3. Is your child able to do everything they want to do even though they have their condition?	121	3.91	0.97	.82	.81	
		4. Does your child feel like everyone else even though they have their condition?	122	3.66	1.07	.75	.82	
		5. Does your child feel free to lead the life they want even though they have their condition?	122	3.76	1.00	.83	.81	
		6. Does your child feel able to do things without you?	122	3.63	0.96	.46	.87	
	Emotion	13. Does your child's condition make them feel bad about themselves?	123	4.01	0.90	.81	.91	0.92
		14. Does your child feel unhappy because of his/her condition?	123	4.00	0.91	.83	.91	
		15. Does your child worry about his/her condition?	123	3.58	0.91	.72	.92	
		16. Does your child's condition make him/her angry?	123	3.49	1.09	.79	.91	
		17. Does your child have fears about the future because of his/her condition?	123	3.72	0.96	.81	.91	
		18. Does your child's condition get him/her down?	123	3.67	0.99	.84	.90	
		19. Does it bother your child that his/her life has to be planned?	123	3.09	1.12	.57	.93	
	Social inclusion	26. Does your child feel that other children/ adolescents understand their condition?	122	3.34	1.16	.30	.83	0.8
		27. Does your child go out with his/her friends?	123	3.92	1.06	.58	.76	
		28. Does your child feel able to play or do things with other children (like sports)?	123	4.20	1.09	.61	.76	
		29. Does your child think that he/she can do most things as well as other children?	123	4.20	0.97	.66	.75	
		30. Does your child feel that their friends enjoy being with them?	122	4.46	0.72	.70	.75	
		31. Does your child find it easy to talk about his/her condition to other people?	123	3.20	1.22	.60	.76	
	Social	20. Does your child feel lonely because of his/her condition?	123	4.13	0.88	.75	.84	0.87
		21. Does your child feel that their teachers behave differently towards them than towards	123	4.02	1.03	.44	.89	
		22. Does your child feel that they have problems concentrating at school because of their	123	4.05	0.97	.62	.86	
		23. Does your child feel that others have something against him/her?	123	4.12	0.92	.79	.83	
		24. Does your child think that others stare at him/her?	123	4.06	1.00	.79	.83	
		25. Does your child feel different from other children/adolescents?	123	3.59	1.14	.73	.84	
	Physical	7. Does your child feel able to run and move as he/she likes?	122	4.21	1.10	.51	.86	0.86
		8. Does your child feel tired because of their condition?	123	3.47	1.18	.70	.83	
		9. Does your child feel that their life is ruled by their condition?	122	3.40	1.11	.77	.81	
		10. Does it bother your child that they have to explain to others what they can and can't do?	123	3.37	1.07	.67	.83	
		11. Does your child find it difficult to sleep because of their condition?	122	4.17	0.94	.46	.87	
		12. Does your child's condition bother them when they play or do other activities?	123	3.54	1.09	.80	.81	
	Treatment	32. Does having to get help with medication from others bother your child?	113	3.70	1.24	.58	.88	0.88
		33. Is it annoying for your child to have to remember his/her medication?	113	3.26	1.15	.79	.84	
		34. Is your child worried about his/her medication?	113	4.10	0.81	.53	.88	
		35. Does taking medication bother your child?	113	2.70	1.17	.79	.84	
		36. Does your child hate taking his/her medicine?	113	3.73	1.12	.69	.86	
		37. Does your child feel that taking medication disrupts his/her everyday life?	113	3.48	1.06	.78	.84	
