# Supplementary material for: Assessing health-related quality of life in Japanese children with a chronic condition: validation of the DISABKIDS chronic generic module
Source: Health Qual Life Outcomes. 2018 May 2;16:85. doi: 10.1186/s12955-018-0911-1 (PMC5932858; doi:10.1186/s12955-018-0911-1)
Supplement: Supplementary file 2 — Summary of Confirmatory Factor Analysis. (PDF 10 kb) [file 12955_2018_911_MOESM2_ESM.pdf]

Appendix 2: Summary of Confirmatory Factor Analysis

Child-report

Domain	Model	CMIN	Df	CMIN/df	GFI	CFI	RMSEA
Mental	Model 0 (original)	100.088	64	1.564	0.874	0.924	0.074
	Model 1 (delete item 6 and 9)	69.708	43	1.621	0.894	0.942	0.078
	Model 2 (error covariance correction model)	**43.668**	**40**	**1.092**	**0.933**	**0.992**	**0.030**
Social	Model 0 (original)	96.321	53	1.817	0.871	0.877	0.090
	Model 1 (error covariance correction model)	**66.542**	**51**	**1.305**	**0.902**	**0.956**	**0.055**
Physical	Model 0 (original)	74.529	53	1.406	0.892	0.941	0.063
	Model 1 (delete item 32)	66.115	43	1.538	0.896	0.936	0.073
	Model 2 (error covariance correction model)	**50.418**	**42**	**1.200**	**0.916**	**0.977**	**0.044**

Parent-report

Domain	Model	CMIN	Df	CMIN/df	GFI	CFI	RMSEA
Mental	Model 0 (original)	125.385	64	1.959	0.856	0.935	0.096
	Model 1 (error covariance correction model)	**76.284**	**61**	**1.251**	**0.909**	**0.984**	**0.049**
Social	Model 0 (original)	137.238	53	2.589	0.824	0.875	0.124
	Model 1 (error covariance correction model)	**82.356**	**51**	**1.615**	**0.884**	**0.953**	**0.077**
Physical	Model 0 (original)	126.862	53	2.394	0.824	0.887	0.116
	Model 1 (error covariance correction model)	**94.866**	**50**	**1.897**	**0.870**	**0.931**	**0.093**
