# Supplementary material for: Assessing health-related quality of life in Japanese children with a chronic condition: validation of the DISABKIDS chronic generic module
Source: Health Qual Life Outcomes. 2018 May 2;16:85. doi: 10.1186/s12955-018-0911-1 (PMC5932858; doi:10.1186/s12955-018-0911-1)
Supplement: Supplementary file 3 — Intercorrelation (Pearson r) between total score and sub-scales for excluding 0 and 100 scores. (PDF 78 kb) [file 12955_2018_911_MOESM4_ESM.pdf]

**Appendix4~~2~~: Correlation coefficients (Pearson r) for total score and sub-scales with the KIDSCREEN for excluding 0 and 100 scores**

| | | Independence | Emotion | Social inclusion | Social exclusion | Physical limitation | Treatment | Total score |
|---|---|---|---|---|---|---|---|---|
| KIDSCREEN | Children | | | | | | | |
| | Self-report version | 0.43 (p=0.002) | 0.49~~52~~ (p=0.002) | 0.51 (p<0.001) | 0.26 (p=0.102) | 0.36 (p=0.014) | 0.46~~1~~ (p=0.04) | 0.38~~60~~ (p=0.007) |
| | Parent version | 0.57 (p<0.001) | 0.45 (p=0.001) | 0.60 (p<0.001) | 0.47 (p=0.001) | 0.48 (p<0.001) | 0.34 (p=0.020) | 0.68 (p<0.001) |
| | Adolescents | | | | | | | |
| | Self-report version | 0.66~~4~~ (p<0.001) | 0.38~~44~~ (p=0.067) | 0.61 (p<0.001) | 0.56 (p<0.001) | 0.60 (p<0.001) | 0.10~~4~~ (p=0.491) | 0.27~~71~~ (p=0.063) |
| | Parent version | 0.64 (p<0.001) | 0.46 (p=0.001) | 0.54 (p<0.001) | 0.46 (p=0.001) | 0.59 (p<0.001) | 0.41 (p=0.004) | 0.68 (p<0.001) |

~~Note: All correlations are significant (P<0.01), except for the coefficient between the sub-scale of Social exclusion and the KIDSCREEN in children, and between the sub-scale of Treatment and the KIDSCREEN in adolescents for self-report.~~
