# Supplementary material for: Assessing health-related quality of life in Japanese children with a chronic condition: validation of the DISABKIDS chronic generic module
Source: Health Qual Life Outcomes. 2018 May 2;16:85. doi: 10.1186/s12955-018-0911-1 (PMC5932858; doi:10.1186/s12955-018-0911-1)
Supplement: Supplementary file 4 — Correlation coefficients (Pearson r) for total score and sub-scales with the KIDSCREEN for excluding 0 and 100 scores. (PDF 97 kb) [file 12955_2018_911_MOESM3_ESM.pdf]

Appendix31: Intercorrelation (Pearson r) between total score and sub-scales for excluding 0 and 100 scores

Self-report/ proxy-report

	Independence	Emotion	Social inclusion	Social exclusion	Physical limitation	Treatment
Children						
Emotion	0.57**/0.58**					
Social inclusion	0.676**/0.55**	0.572**/0.32*				
Social exclusion	0.7166**/0.64**	0.66**/0.60**	0.56**/0.49**			
Limitation	0.5748**/0.60**	0.625**/0.66**	0.53**/0.35**	0.75**/0.72**		
Treatment	0.1921/0.001	0.4037*/0.42**	-0.021/0.18	-0.063/0.30*	0.0613/0.32*	
Total score	0.773**/0.75**	0.878**/0.84**	0.761**/0.55**	0.821**/0.83**	0.874**/0.83**	0.3241**/0.53**
Adolescents						
Emotion	0.652**/0.71**					
Social inclusion	0.52**/0.43**	0.353**/0.21				
Social exclusion	0.596**/0.53**	0.69**/0.57**	0.47**/0.56**			
Limitation	0.6055**/0.71**	0.612**/0.76**	0.52**/0.49**	0.74**/0.77**		
Treatment	0.096/0.47**	0.1825/0.62**	0.050/0.08	0.29127/0.35*	0.134/0.45**	
Total score	0.8076**/0.84**	0.7881**/0.87**	0.7068**/0.60**	0.821**/0.80**	0.876**/0.89**	0.3441**/0.65**

*P<0.05, **P<0.01
